# Arguing for Adaptive Clinical Trials in Sepsis

**DOI:** 10.3389/fimmu.2018.01502

**Published:** 2018-06-28

**Authors:** Victor B. Talisa, Sachin Yende, Christopher W. Seymour, Derek C. Angus

**Affiliations:** Clinical Research, Investigation, and Systems Modeling of Acute Illness Center, Department of Critical Care Medicine, University of Pittsburgh, Pittsburgh, PA, United States

**Keywords:** sepsis, adaptive clinical trials, Bayesian statistics, platform trials, response adaptive randomization

## Abstract

Sepsis is life-threatening organ dysfunction due to dysregulated response to infection. Patients with sepsis exhibit wide heterogeneity stemming from genetic, molecular, and clinical factors as well as differences in pathogens, creating challenges for the development of effective treatments. Several gaps in knowledge also contribute: (i) biomarkers that identify patients likely to benefit from specific treatments are unknown; (ii) therapeutic dose and duration is often poorly understood; and (iii) short-term mortality, a common outcome measure, is frequently criticized for being insensitive. To date, the majority of sepsis trials use traditional design features, and have largely failed to identify new treatments with incremental benefit over standard of care. Traditional trials are also frequently conducted as part of a drug evaluation process that is segmented into several phases, each requiring separate trials, with a long time delay from inception through design and execution to incorporation of results into clinical practice. By contrast, adaptive clinical trial designs facilitate the evaluation of several candidate treatments simultaneously, learn from emergent discoveries during the course of the trial, and can be structured efficiently to lead to more timely conclusions compared to traditional trial designs. Adoption of new treatments in clinical practice can be accelerated if these trials are incorporated in electronic health records as part of a learning health system. In this review, we discuss challenges in the evaluation of treatments for sepsis, and explore potential benefits and weaknesses of recent advances in adaptive trial methodologies to address these challenges.

## Introduction

Sepsis is a leading cause of critical illness and mortality globally (1, 2). It is a clinical syndrome and defined as a dysregulated host response to infection resulting in organ failure (3). This definition implies that different combinations of host and pathogen characteristics and interactions among them may lead to the same clinical picture. This inherent heterogeneity presents a major challenge to develop treatments and may be an important reason for recent neutral clinical trials. In addition, the traditional sequence to develop new therapeutics by pharmaceutical companies has several limitations that may exacerbate these challenges, and lead to prolonged evaluation periods of up to a decade before results can be operationalized (4). In this review, we discuss challenges in the evaluation of treatments for sepsis, and explore potential benefits of recent advances in adaptive trial methodologies to address these challenges.

## Challenges in the Evaluation of Potential Treatments

### Sepsis Is Extremely Heterogeneous

Several preclinical models suggest that sepsis results from a disproportionate pro-inflammatory response to infection. However, several clinical trials of anti-inflammatory agents in relatively broad patient populations were disappointing (5). Subsequent findings indicated that circulating levels of pro-inflammatory cytokines, such as IL-6 and TNF ranged from 8 to 1,550,000 pg/ml and 7 to 57,000 pg/ml, respectively, in patients with sepsis (6). Thus, the assumption that all patients would benefit equally from anti-inflammatory agents is unlikely. The host immune response during sepsis is complex and dynamic, involving excessive pro-inflammation and immunosuppression, often concomitantly. The balance of pro- and anti-inflammatory responses also evolves over the course of illness, and may be prognostic (7, 8).

A sustained dysregulated immune response may lead to profound alterations in the endothelium and surrounding tissues, including increased leukocyte adhesion, coagulation, and vasodilation, and loss of barrier function, hypoperfusion, and tissue hypoxemia (9). These disruptions and others lead to multisystem organ dysfunction, including acute kidney injury, neurologic complications, acute respiratory distress syndrome (ARDS), hepatic failure, and shock. However, the specific organ systems affected varies between patients. This organ failure is commonly seen against a backdrop of multimorbidity, a condition where two or more comorbidities may exist in a patient. Multimorbidity is observed in more than 30% of ICU patients (10) and further increases clinical heterogeneity. Taken as a whole, such complex variability stemming from these and other sources (e.g., host genetics and microbiologic factors) poses significant challenges to the efficient design and conduct of clinical trials, particularly those testing interventions targeting a specific mechanism. Some subsets of patients may benefit from such an intervention, while others may result in no benefit or even harm. For example, in simulated trials of anti-TNF studied *in silico*, benefit was observed after considering genetic and microbiological factors (11). However, most trials of anti-TNF in humans have ignored these factors.

### Biomarkers That Predict Treatment Response Are Unknown Before Initiating a Trial

As discussed above, sepsis pathobiology is complex and evolving. As opposed to other diseases in which the natural history and risk factors are better understood, there are critical gaps in our knowledge of the potential markers of prognosis after sepsis (i.e., prognostic markers) and markers that predict treatment response (i.e., predictive markers). This distinction between prognostic and predictive markers is critical. For example, in a trial of anti-TNF, a group of patients with high levels of IL-6 had a higher mortality rate, but not a higher drug response, suggesting prognostic but not predictive utility as a biomarker (6). In scenarios in which a treatment’s effect is meaningfully heterogeneous among the patient population, predictive markers are useful in explaining the sources of this heterogeneity of treatment effect (HTE). Without knowledge of the drivers of HTE, researchers and drug companies either ignore heterogeneity and enroll broadly or take a leap of faith and enroll narrowly based on suspected predictive biomarkers. If the biomarkers are not validated, the latter scenario could lead to exclusion of patients who would have benefited, or inclusion of patients who will not respond to the treatment.

While it is possible that single markers may be identified as sufficiently predictive by themselves, it is also possible that groups of genetic, metabolic, and/or clinical features may often occur together, forming groups or “phenotypes” of patients within the broad umbrella of sepsis that may have similar outcomes or treatment response rates. In sepsis and septic shock, phenotypes have recently been described that are associated with variable risk of mortality and would be considered “prognostic” (12, 13). In ARDS, phenotypes are described and found to respond variably to different fluid and ventilator management strategies (14). As sepsis phenotypes are further described, it will be important to allow for future trials to incorporate possible markers of HTE as efficiently as possible to avoid missing a true drug response by enrolling too broadly or too narrowly.

### Optimal Therapeutic Dose and Duration Is Often Poorly Understood

The selection of treatment dosing and duration is often based on limited animal studies and small pharmacokinetic and pharmacodynamics studies in humans, with a focus on evaluating safety (15). Preclinical studies are commonly carried out in simple, often healthy and young, rodent models exposed to a specific endotoxin or using the cecal-ligation and puncture model. These models are criticized for bearing little relation to human sepsis, which occurs in older patients with significant comorbidities and who are often receiving adjuvant support. Furthermore, treatment in the rodent models has typically coincided with the timing of the infectious challenge; in humans, time between treatment and the initial infection is unknown and likely variable (5). The pitfalls of designing a phase 3 study based on an optimistic interpretation of preclinical and traditional early phase designs are suggested in the case of nitric oxide synthase inhibitor *N*^G^-monomethyl-l-arginine (L-NMMA). Promoted by encouraging preclinical data, a phase 2 safety study was conducted and showed a promising trend toward increased survival in the treated group (16). However, a subsequent phase 3 trial using a similar dosing strategy found increased mortality in the treatment arm overall, but significant survival benefit in a group with a relatively low exposure to the drug (17). The investigators concluded that this was likely a result of a relatively high exposure to the drug overall. Other studies suggest that in some cases dosing and duration may interact with each other in complex ways (11). Thus, the impact of dosing and duration, and their interaction, on sepsis outcomes is often poorly understood prior to initiation of phase 3 studies.

### Short-Term Mortality Is Insensitive as a Primary Outcome

Short-term, all-cause mortality is a commonly used primary endpoint in phase 2 and 3 trials (18). However, short-term mortality is declining (19, 20), and those who do not die early often die in the ensuing months or incur considerable morbidity (21, 22). Moreover, pure mortality endpoints are criticized for being insensitive measures of biologic activity, and thus poor tools for use in early phase trials for selection of dosing and duration. Recognizing these shortcomings, there is interest in identifying and validating short-term endpoints that are both more sensitive to treatment effects and good proxies for longer-term patient centered outcomes; one proposed alternative is combination of mortality and organ support duration (23).

## Novel Trial Methodologies and their Utility for Evaluation of Sepsis Treatments

Most sepsis trials use traditional design features, in which all trial parameters are fixed for the duration of the study, including randomization ratios, sample sizes, number of treatment arms, and inclusion/exclusion criteria, among others. These designs have the advantage of optimal statistical power and internal validity when there are only two treatment arms, but this comes at the expense of flexibility should the investigator be interested in testing more complex and potentially numerous hypotheses (24).

By contrast, adaptive designs facilitate the evaluation of several research questions simultaneously and embrace the possibility of emergent discoveries during the course of the trial. During an adaptive trial, updates are made to the design parameters following interim analysis, often conducted several times before the trial’s completion. The decision rules dictating which updates can be made are predetermined before initiation to avoid introducing bias (25–27). Below we discuss several features of adaptive designs that could theoretically be used to address key challenges in the evaluation of sepsis treatments (Table 1, section A). In addition, we discuss ways in which adaptive designs can potentially accelerate the drug evaluation process (summarized in the Table 1, section B).

**Table 1:** Comparison of traditional and adaptive design features in addressing challenges of sepsis to the evaluation of beneficial treatments (section A), and ways in which common features of each influence the total time spent evaluating treatments (section B).

	Traditional trial designs	Adaptive trial designs
**Section A. Challenges of sepsis to the evaluation of beneficial treatments**		

High degree of disease heterogeneity as a result of variability among patients (e.g., biochemical and genetic), within patients (e.g., temporal dynamics of immune response), and infection characteristics (e.g., site and pathogen)	Usually test a single drug in a single predefined population; usually use 1:1 ratio of randomization to experimental and control arms	Response adaptive randomization (RAR) enables multiple drugs to be tested in potentially different subgroups based on projected mechanism of action, while preserving efficiency. Randomized, embedded, multifactorial adaptive platform enables recruitment from as broad a population base as possible, necessary for sample sizes to satisfy complex designs testing drugs in multiple subgroups

Specific biomarker profiles may predict treatment response, but the optimal sub-populations are unknown	Usually restricted to a single, predefined population, and as a result, enrollment criteria are often too broad or too narrow	Enrichment designs enable identification of the sub-population in which treatment response is optimized over the course of the trial

Optimal therapeutic dose and duration is often poorly understood	Due to trial inefficiencies, dose selection is often under-studied, potentially including under- or overdosing or using a dose that is constant despite variable patient requirements	Dose-finding designs can use RAR to study optimal dosing while preserving efficiency; can open higher dose arms as evidence in lower doses accumulates in support of efficacy and safety

Short-term mortality is the accepted clinical endpoint, but has been criticized insensitive to possible drug-related changes in morbidity and long-term mortality	A single primary endpoint is usually fixed before the start of the trial	Platform designs can be leveraged to evaluate proxy endpoints over time and feed this information back into the trial by incorporating it into the RAR algorithm

**Section B. Major contributors to total time spent evaluating drugs**		

Drug evaluation machinery	New study sites, protocols, and designs are usually established anew for each drug	Platform designs can include perpetually active master protocols that facilitate continuous use of existing trial resources on selection of drugs that is periodically updated

Number of drug arms tested simultaneously	Usually one. Traditional trials are most efficient when testing a single drug against placebo. Testing multiple drugs requires larger sample compared with adaptive designs	Multiple drugs can be compared to a single placebo arm while maintaining statistical efficiency using RAR, obviating the need for separate trials

Transitions between phases of the drug evaluation process	Phases are usually carried out one at a time, with sometimes long intervals in between for design and approval of the next phase	Seamless designs consolidate multiple phases into a single protocol that is designed, approved, and executed as a single trial. Sample sizes for component phases can be smaller if efficacy in the final phase is estimated using data from all phases

### Bayesian Response Adaptive Randomization (RAR)

Although it is optimal to conduct a two-arm trial using a traditional design, such approaches are inefficient when evaluating more than two treatments against control. Traditional designs typically use the frequentist statistical paradigm, where prior information about efficacy is utilized formally only in the design of a clinical trial (e.g., power calculations), but not during analyses. Alternatively, Bayesian statistical approaches provide a formal mathematical mechanism for combining prior and current information for use in the design, conduct, and final analysis stages of the trial (28). Adaptive trial designs have been developed under both frequentist and Bayesian paradigms (29).

The Bayesian paradigm provides a natural foundation for statistical tools utilized in many adaptive designs, which involve iteratively updating or “adapting” information gathered during the trial (25). One such tool, RAR, is used to increase efficiency when testing more than one treatment against control. Over the course of the trial, accumulating data are used to adjust the randomization probabilities to preferentially assign future patients to better-performing treatment arms (26). Typically, the first block of patients are randomized to each arm in equal proportion and randomization probabilities for subsequent blocks are calculated based on information accumulated prior to starting the block. A common way of executing RAR is by calculating the Bayesian predictive probability that a given treatment arm will be superior to control in the final analysis. This calculation often requires sophisticated computer simulations, but effectively integrates not only uncertainty about the true drug benefit based on data accumulated so far but also uncertainty about future data that have not yet been observed (30). Unless the predictive probability is too low (i.e., the arm should be dropped), or sufficiently high (the arm may “graduate” to the next phase of testing), the updated randomization probability for the next block of patients is proportional to the predictive probability of success for the treatment relative to control (27). Frequentist adaptive trial designs exist, but are not amenable to RAR.

Implementation of RAR could benefit sepsis trials in several ways. First, it would enable the study of multiple drugs simultaneously in a phase 2 trial, increasing the chances that at least one drug being tested will improve outcomes while reducing the time and costs needed to evaluate them individually by “learning” which ones are superior during the phase 2 trial and will have high likelihood of success in future phase 3 trials. The use of RAR instead of fixed randomization ratios underscores a focus on identifying the best-performing arm, instead of expending resources to rank all arms from worst to best performance. Second, instead of using RAR to assign patients to different arms, phase 2 adaptive trials could test different dosing and/or duration strategies for a single drug to better inform the optimal treatment strategy for phase 3 testing. This approach was implemented in SEPSIS-ACT, an adaptive trial of selepressin dosing strategies in adults with septic shock (31). In this trial, RAR was used to allocate patients to three dose levels until predefined checkpoints for safety and efficacy were triggered. If necessary, a fourth could be introduced based on response to the three doses.

### Adaptive Enrichment Designs

Often there is interest in a variety of drugs as well as identifying potential sub-populations within which the drugs are most effective. In a traditional enrichment design, randomization is simply limited to patients with a specific biomarker profile known to be predictive of treatment response. However, we may not know which patient groups may benefit the most from a treatment in sepsis. Using adaptive trial methodologies, it is possible to incorporate putative predictive biomarkers to “learn” the optimal biomarker profile in the case that a meaningful underlying HTE exists (32).

In one approach, the RAR algorithm is used in an adaptive platform trial (see below) of several drugs, where patients are categorized into several candidate predictive biomarker strata before randomization. In the BATTLE I trial, non-small cell lung cancer patients were classified into four candidate strata defined by genomic and expression markers before being randomized to one of four drug regimens. Separately for each stratum, the RAR weights were adjusted as data accumulated to favor assignment of drugs with higher within-stratum response rates (33). The results from BATTLE 1 both confirmed pre-specified hypotheses of treatment efficacy in the presence of individual markers related to the treatments’ mechanism of action, and also suggested new treatment–biomarker interactions (34).

An alternative enrichment approach allows for more flexibility in the scenario where candidate predictive biomarkers have not been identified. In this framework, the optimal target population for the experimental treatment is adaptively learned and estimated as a function of baseline covariates (35, 36). Such designs could be useful, for example, to identify the optimal threshold value of a predictive biomarker to use for splitting the patient population into responsive and non-responsive strata (35).

While underlying drug response strata may exist and may be delineated by putative biomarkers, demonstrating this may be difficult in scenarios where treatment effects are relatively homogeneous or when the overall treatment effect is small. Thus, adaptive enrichment strategies present a potential advantage by incorporating mechanisms to adapt to the presence of HTE if evidence for it mounts over the course of the trial.

### Seamless Designs

The traditional drug evaluation pipeline is usually segmented into several phases, each involving a brand new trial. To streamline this process and reduce associated time and costs, a number of designs have been developed that combine multiple phases into a single trial, including several within the Bayesian adaptive framework (37). This approach was implemented into the SEPSIS-ACT trial, an adaptive phase 2b/3 trial (31). Part 1 of SEPSIS-ACT uses RAR to “learn” which dosing regimen leads to greatest efficacy, while part 2 is a confirmation stage randomizing 1,000 new patients equally between control and a single treatment arm featuring the dose selected in part 1. Early stopping of part 1 would occur if enough evidence had been obtained to select an optimal dose; otherwise enrollment would continue up to a predetermined maximum sample size. To further increase efficiency, all data from parts 1 and 2 are incorporated in the final analysis. Thus, adaptive seamless designs may lead to more timely conclusions, an advantage which is just as useful for patients and researchers in the case of a truly effective treatment as for a truly ineffective one.

### Adaptive Platform Trials

There is significant effort required to launch a trial, including preparing trial documents, identifying sites, initiating the trial, and obtaining regulatory approval. As the name suggests, the adaptive platform trial is capable of being a *platform* for testing experimental treatments in a perpetual manner *via* a common master protocol, by dropping treatments lacking efficiency and adding new treatments going into the future. They are able to incorporate several design features of adaptive trials, such as RAR, biomarker enrichment, and seamless transitioning, often all in the same design. Currently there are platform trials enrolling patients in oncology (38, 39), infectious diseases (40), neurology (41), and intensive care (42).

**Figure 1:**
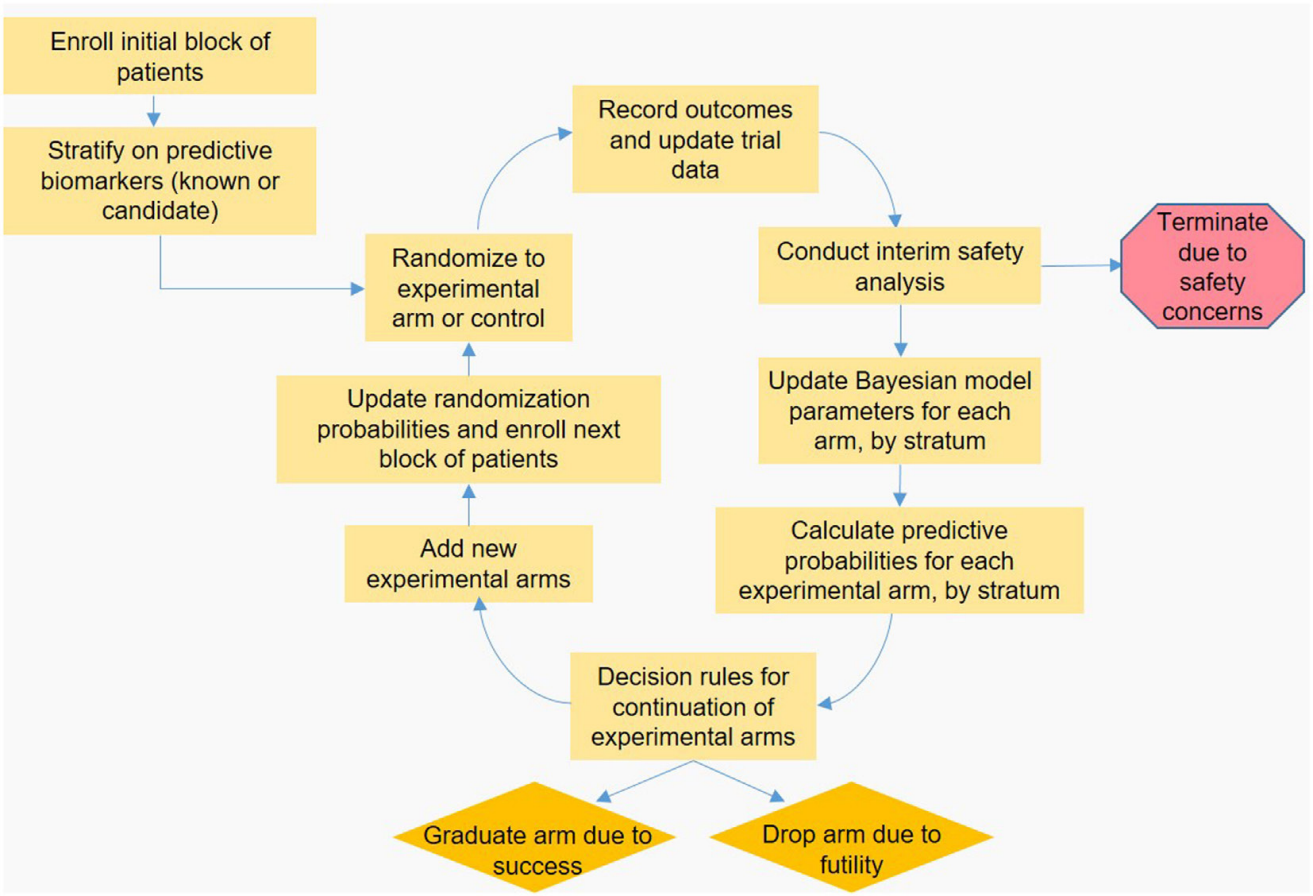
Schematic representation of a hypothetical adaptive platform trial. An initial block of patients is stratified based on known or candidate predictive biomarkers, and then randomized to an experimental or control arm. Once a predefined number of patients is enrolled, outcomes are observed and the data are input to the Bayesian statistical model by arm and stratum, which is used to calculate the predictive probabilities (PP) that each experimental arm will be superior to control in the final analysis. These PP are checked against predefined decision boundaries established so that arms with poor probability of success are dropped, and arms with high probability of success “graduate” to the next phase of testing. Arms with PP that do not require dropping or graduation continue enrolling subjects; arms that are removed may be replaced by new experimental treatments, accrual permitting. Finally, the PP are used to update randomization probabilities used for the next block of patients to be enrolled, and the feedback loop begins anew.

In a platform trial, the feedback loop involving collecting data, updating the Bayesian statistical model and updating RAR weights is modified to enable new arms to be added, and old arms to either be dropped or “graduate” to the next phase of testing. A schematic of the platform trial design is shown in the Figure 1. I-SPY 2 is a phase 2 platform trial in women with locally advanced breast cancer, and out of eight treatments entered into the trial loop so far, two are considered promising enough to “graduate” out of the trial (43, 44). GBM-AGILE, an inferentially seamless phase 2/3 platform trial in glioblastoma, was designed so that “graduating” treatments are seamlessly transitioned into phase 3 confirmatory testing (39). Both I-SPY 2 and GBM-AGILE incorporate enrichment biomarkers hypothesized to be predictive of response for specific treatment arms.

There is considerable pressure to identify short-term endpoints that can be used to speed the evaluation of treatments by accurately predicting treatment response in terms of a gold-standard endpoint, such as long-term mortality. I-SPY 2 and GBM-AGILE both leverage accumulating data in a continually updated Bayesian longitudinal model to generate predictions of the long-term endpoints for use in updating RAR weights (26, 30, 45). In I-SPY 1, for example, it was found that MRI outcomes within the first few weeks following treatment predicted pathological complete response (pCR) at the time of surgery, about 5 months after treatment (46). Thus, short-term MRI data are used to predict pCR in I-SPY 2 for the purposes of updating the RAR weights months before the actual pCR data are observed, increasing efficiency (38, 46). In GBM-AGILE, useful proxy endpoints are being learned and vetted within the trial, and potentially different endpoints are expected to capture the effects of different treatments (39, 45).

The incorporation of Bayesian models in adaptive sepsis trials could theoretically provide a means of evaluating how changes in short-term endpoints (e.g., 28-day organ failure free days) due to treatment correspond with changes in new long-term endpoints such as quality-adjusted life-years at 6 months.

### Embedded, Multifactorial Adaptive Platform Trials

Randomized, embedded, multifactorial adaptive platform (REMAP) trials utilize all of the features of a perpetual adaptive platform trials like I-SPY 2 or GBM-AGILE, the key distinction being that a REMAP trial is executed directly within clinical practice through the electronic medical record [EMR (47)]. A key advantage of embedding trials in clinical care is to create a “learning health system” by enrolling most eligible participants, which increases the speed with which new knowledge is generated and implemented in routine clinical care. In addition, it maximizes internal and external validity, and minimizes operational complexity at the bedside (there is no need to distinguish between trial and non-trial patients, because all patients are trial patients). While screening and recruitment for a REMAP can be conducted by research staff, it is not intended that recruitment should be dependent on research staff because they are typically present during office hours. Thus, REMAP trials may reduce costs.

Most REMAP trials determine the effectiveness of various treatments used in routine clinical care but in a randomized setting. An example is the REMAP-CAP trial being conducted in patients with community-acquired pneumonia severe enough to be admitted to an intensive care unit (42). Upon submitting treatment orders through the EMR, the clinician can choose to instead be randomized the most promising treatment regimens (utilizing RAR weights). To capture the clinical complexity of treatment plans involving combinations of treatments, patients are randomized to multiple sets of treatments within different domains. For instance, different antibiotic regimens and immunomodulatory drugs may be compared in patients with severe pneumonia. This complexity not only requires the use of complex statistical models incorporating interaction terms but also increases penetration of the trial through its embedment in the EMR. Like I-SPY 2 and GMB-AGILE, REMAP-CAP is designed to be perpetual, and as such include mechanisms to incorporate control arms that are updated to incorporate any newly discovered standards of care, e.g., resulting from the trial itself. Although not implemented yet in REMAP-CAP, there is also the capability to incorporate enrichment biomarkers as well. Investigators have recently been funded to launch REMAP initiatives in other conditions, including anti-microbial resistance, cystic fibrosis, hepatitis C, and operative stress in the elderly.

## Inherent Challenges of Adaptive Designs

Adaptive designs may have many promising features for future trials in sepsis, but they also come with their own challenges. Statistical models and the exploration of operating characteristics are complex and simulation-intensive. Selection of potential trial trajectories is especially important during the simulation process, as an overly narrow set of scenarios may lead researchers to fail to understand the consequences of their design choices. A range of alternative trajectories should be explored by varying important simulation parameters, for example: choice of Bayesian prior (assuming a Bayesian model is used); choice of data model; underlying treatment effects for each arm; proportions of patients within subtypes; accrual rates; and others. For designs relying on simulation-based outcome metrics such as the predictive probability of success, failure to explore sensitivity to modeling assumptions may pose risk to future patients, e.g., if drugs are erroneously selected for graduation. In designs considering many subgroups or combinations of drugs, careful consideration must be taken to craft statistical models with only the necessary complexity to preserve statistical power. In addition, the benefits to trial efficiency of periodically updating RAR weights are somewhat dependent on patient accrual rates; if accrual of data occurs faster compared to ideal updating time, RAR updates may occur based on incomplete follow-up. The complexity of these designs also creates difficulties communicating their features to important stakeholders who may be unfamiliar with them, such as funding agencies, institutional review boards, patients, research journals, and clinicians.

## Conclusion

Many studies employing Bayesian adaptive trial designs have already led to promising discoveries in several diseases, including the identification of two promising candidate drugs in breast cancer. These studies leverage several key features of adaptive designs, including RAR, enrichment methods, seamless transitioning between trial phases, perpetual platforms for ongoing evaluation of candidate treatments, integration within the EMR, and others. These features are well suited to address the many challenges presented by complex, heterogeneous diseases, yet are rarely utilized in sepsis. Adoption of these designs may aid in the efficient identification of promising treatments for sepsis.

## Author Contributions

All authors contributed to the editing and preparation of this manuscript.

## Conflict of Interest Statement

The authors declare that the research was conducted in the absence of any commercial or financial relationships that could be construed as a potential conflict of interest.

## References

[1] Vincent J-L Marshall JC Namendys-Silva SA Francois B Martin-Loeches I Lipman J Assessment of the worldwide burden of critical illness: the intensive care over nations (ICON). Lancet Respir Med (2014) 2:380–6. 10.1016/S2213-2600(14)70061-X 24740011

[2] Fleischmann C Scheraq A Adhikari NK Hartog CS Tsaganos T Schlattmann P Assessment of global incidence and mortality of hospital-treated sepsis. Current estimates and limitations. Am J Respir Crit Care Med (2016) 193:259–72. 10.1164/rccm.201504-0781OC 26414292

[3] Singer M Deutschman CS Seymour CW Shankar-Hari M Annane D Bauer M The third international consensus definitions for sepsis and septic shock (SEPSIS-3). JAMA (2016) 315:801–10. 10.1001/jama.2016.0287 26903338 PMC4968574

[4] Rhodes A Evans LE Alhazzani W Levy MM Antonelli M Ferrer R Surviving Sepsis Campaign: International guidelines for management of sepsis and septic shock 2016. Crit Care Med (2017) 45(3):486–552. 10.1097/CCM.0000000000002255 28098591

[5] Marshall JC. Why have clinical trials in sepsis failed? Trends Mol Med (2014) 20:195–203. 10.1016/j.molmed.2014.01.007 24581450

[6] Panacek EA Marshall JC Albertson TE Johnson DH Johnson S MacArthur RD Efficacy and safety of the monoclonal anti-tumor necrosis factor antibody F(ab’)2 fragment afelimomab in patients with severe sepsis and elevated interleukin-6 levels. Crit Care Med (2004) 32:3173–82. 10.1097/01.CCM.0000145229.59014.6C 15640628

[7] Kellum JA Kong L Fink MP Weissfeld LA Yealy DM Pinsky MR Understanding the inflammatory cytokine response to pneumonia in sepsis: results of the genetic and inflammatory markers of sepsis (GenIMS) study. Arch Intern Med (2007) 167:1655–63. 10.1001/archinte.167.15.1655 17698689 PMC4495652

[8] Boomer JS To K Chang KC Takasu O Osborne DF Walton AH Immunosuppression in patients who die of sepsis and multiple organ failure. JAMA (2011) 306:2594–605. 10.1001/jama.2011.1829 22187279 PMC3361243

[9] Gotts JE Matthay MA. Sepsis: pathophysiology and clinical management. BMJ (2016) 353:i1585. 10.1136/bmj.i1585 27217054

[10] Christiansen CF Christensen S Johansen MB Larsen KM Tonnesen E Sorensen HT. The impact of pre-admission morbidity level on 3-year mortality after intensive care: a Danish cohort study. Acta Anaesthesiol Scand (2011) 55:962–70. 10.1111/j.1399-6576.2011.02480.x 21770901

[11] Clermont G Bartels J Kumar R Constantine G Vodovotz Y Chow C. In silico design of clinical trials: a method coming of age. Crit Care Med (2004) 32:2061–70. 10.1097/01.CCM.0000142394.28791.C3 15483415

[12] Wong HR Sweeney TE Hart KW Khatri P Lindsell CJ. Pediatric sepsis endotypes among adults with sepsis. Crit Care Med (2017) 45:e1289–91. 10.1097/CCM.0000000000002733 28991828 PMC5693699

[13] Scicluna BP van Vught LA Zwinderman AH Wiewel MA Davenport EE Burnham KL Classification of patients with sepsis according to blood genomic endotype: a prospective cohort study. Lancet Respir Med (2017) 5:816–26. 10.1016/S2213-2600(17)30294-1 28864056

[14] Famous KR Delucchi K Ware LB Kangelaris KN Liu KD Thompson BT Acute respiratory distress syndrome subphenotypes respond differently to randomized fluid management strategy. Am J Respir Crit Care Med (2017) 195:331–8. 10.1164/rccm.201603-0645OC 27513822 PMC5328179

[15] Van der Poll T van de Veerdonk FL Scicluna BP Netea MG. The immunopathology of sepsis and potential therapeutic targets. Nat Rev Immunol (2017) 17:407–20. 10.1038/nri.2017.36 28436424

[16] Bakker J Grover R McLuckie A Holzapfel L Andersson J Lodato R Administration of the nitric oxide synthase inhibitor NG-methyl-L-arginine hydrochloride (546C88) by intravenous infusion for up to 72 hours can promote the resolution of shock in patients with severe sepsis: results of a randomized, double-blind, placebo-controlled multicenter study (study no. 144-002). Crit Care Med (2004) 32:1–12. 14707554 10.1097/01.CCM.0000105118.66983.19

[17] Lopez A Lorente JA Steingrub J Bakker J McLuckie A Willatts S Multiple-center, randomized, placebo-controlled, double-blind study of the nitric oxide synthase inhibitor 546C88: Effect on survival in patients with septic shock. Crit Care Med (2004) 32:21–30. 10.1097/01.CCM.0000105581.01815.C6 14707556

[18] Marshall JC Vincent JL Guyatt G Angus DC Abraham E Bernard G Outcome measures for clinical research in sepsis: a report of the 2nd Cambridge Colloquium of the International Sepsis Forum. Crit Care Med (2005) 33:1708–16. 10.1097/01.CCM.0000174478.70338.03 16096445

[19] Kaukonen KM Bailey M Suzuki S Pilcher D Bellomo R. Mortality related to severe sepsis and septic shock among critically Ill patients in Australia and New Zealand, 2000-2012. JAMA (2014) 311:1308–16. 10.1001/jama.2014.2637 24638143

[20] Rhee C Dantes R Epstein L Murphy DJ Seymour CW Iwashyna TJ Incidence and trends of sepsis in US hospitals using clinical vs claims data, 2009–2014. JAMA (2017) 318:1241–9. 10.1001/jama.2017.13836 28903154 PMC5710396

[21] Liu V Lei X Prescott HC Kipnis P Iwashyna TJ Escobar GJ. Hospital readmission and healthcare utilization following sepsis in community settings. J Hosp Med (2014) 9:502–7. 10.1002/jhm.2197 24700730 PMC4241549

[22] Prescott HC Osterholzer JJ Langa KM Angus DC Iwashyna TJ. Late mortality after sepsis: propensity matched cohort study. BMJ (2016) 353:i2375. 10.1136/bmj.i2375 27189000 PMC4869794

[23] Young P Hodgson C Dulhunty J Saxena M Bailey M Bellomo R End points for phase II trials in intensive care: recommendations from the Australian and New Zealand Clinical Trials Group consensus panel meeting. Crit Care Resusc (2012) 14:211–5. 22963216

[24] Berry SM Connor JT Lewis RJ The platform trial: an efficient strategy for evaluating multiple treatments. JAMA (2015) 313:1619–920. 10.1001/jama.2015.2316 25799162

[25] Spiegelhalter DJ Freedman LS Parmar MKB Bayesian approaches to randomized trials. JRSSA (1994) 157:357–416. 10.2307/2983527

[26] Berry DA. Bayesian clinical trials. Nat Rev Drug Discov (2006) 5:27–36. 10.1038/nrd1927 16485344

[27] Berry DA Adaptive clinical trials in oncology. Nat Rev Clin Oncol (2012) 9:199–207. 10.1038/nrclinonc.2011.165 22064459

[28] Gelman A Carlin JB Stern HS Dunson DB Vehtari A Rubin DB Bayesian Data Analysis. 3rd ed Boca Raton, FL: CRC Press (2014).

[29] Park JJ Thorlund K Mills EJ. Critical concepts in adaptive clinical trials. Clin Epidemiol (2018) 10:343–51. 10.2147/CLEP.S156708 29606891 PMC5868584

[30] Saville BR Connor JT Ayers GD Alvarez J. The utility of Bayesian predictive probabilities for interim monitoring of clinical trials. Clin Trials (2014) 11:485–93. 10.1177/1740774514531352 24872363 PMC4247348

[31] Lewis RJ Angus DC Laterre PF Kjølbye AL van der Meulen E Blemings A Rationale and design of an adaptive phase 2b/3 clinical trial of selepressin for adults in septic shock. Ann Am Thorac Soc (2018) 15:250–7. 10.1513/AnnalsATS.201708-669SD 29388815

[32] Simon R. Critical review of umbrella, basket, and platform designs for oncology clinical trials. Clin Pharmacol Ther (2017) 102:934–41. 10.1002/cpt.814 28795401

[33] Liu S Lee JJ. An overview of the design and conduct of the BATTLE trials. Chin Clin Oncol (2015) 4:33–46. 10.3978/j.issn.2304-3865.2015.06.07 26408300

[34] Kim ES Herbst RS Wistuba II Lee JJ Blumenschein GR Jr Tsao A The BATTLE trial: personalizing therapy for lung cancer. Cancer Discov (2011) 1:44–53. 10.1158/2159-8274.CD-10-0010 22586319 PMC4211116

[35] Simon N Simon R. Adaptive enrichment designs for clinical trials. Biostatistics (2013) 14:613–25. 10.1093/biostatistics/kxt010 23525452 PMC3769998

[36] Simon N Simon R. Using Bayesian modeling in frequentist adaptive enrichment designs. Biostatistics (2018) 19:27–41. 10.1093/biostatistics/kxw054 28520893 PMC6075009

[37] Zang Y Lee JJ Adaptive clinical trial designs in oncology. Chin Clin Oncol (2014) 3(4):49 10.3978/j.issn.2304-3865.2014.06.04 PMC4369921 25811018

[38] Barker AD Sigman CC Kelloff GJ Hylton NM Berry DA Esserman LJ. I-SPY2: an adaptive breast cancer trial design in the setting of neoadjuvant chemotherapy. Clin Pharmacol Ther (2009) 86:97–100. 10.1038/clpt.2009.68 19440188

[39] Alexander BM Ba S Berger MS Berry DA Cavenee WK Chang SM Adaptive global innovative learning environment for glioblastoma: GBM AGILE. Clin Cancer Res (2018) 24:737–43. 10.1158/1078-0432.CCR-17-0764 28814435

[40] Berry SM Petzold EA Dull P Thielman NM Cunningham CK Corey GR A response adaptive randomization platform trial for efficient evaluation of Ebola virus treatments: a model for pandemic response. Clin Trials (2016) 13:22–30. 10.1177/1740774515621721 26768569 PMC5583707

[41] Ritchie CW Molinuevo JL Truyen L Satlin A Van der Geyten S Lovestone S Development of interventions for the secondary prevention of Alzheimer’s dementia: the European Prevention of Alzheimer’s Dementia (EPAD) project. Lancer Psychiatry (2015) 3:179–86. 10.1016/S2215-0366(15)00454-X 26683239

[42] REMAP-CAP: Randomized Embedded Multifactorial Adaptive Platform Trial in Community Acquired Pneumonia. (2016). Available from: https://clinicaltrials.gov/ct2/show/NCT02735707 (Accessed: April 30, 2018).

[43] Park JW Liu MC Yee D Yau C van’t Verr LJ Symmans WF Adaptive randomization of neratinib in early breast cancer. N Engl J Med (2016) 375:11–22. 10.1056/NEJMoa1513750 27406346 PMC5259558

[44] Rugo HS Olopade OI DeMichele A Yau C van ’t Veer LJ Buxton MB. Adaptive randomization of veliparib-carboplatin treatment in breast cancer. N Engl J Med (2016) 375:23–34. 10.1056/NEJMoa1513749 27406347 PMC5259561

[45] Trippa L Wen PY Parmigiani G Berry DA Alexander BM. Combining progression-free survival and overall survival as a novel composite endpoint for glioblastoma trials. Neuro Oncol (2015) 17:1106–13. 10.1093/neuonc/nou345 25568226 PMC4490868

[46] Mukhtar RA Yau C Rosen M Tandon VJ The I-Spy 1 TRIAL and ACRIN 6657 Investigators Hylton N Clinically meaningful tumor reduction rates vary by prechemotherapy MRI phenotype and tumor subtype in the I-SPY 1 TRIAL (CALGB 150007/150012; ACRIN 6657). Ann Surg Oncol (2013) 20:3823–30. 10.1245/s10434-013-3038-y 23780381 PMC3824937

[47] Angus DC Fusing randomized trials with big data: the key to self-learning health care systems? JAMA (2015) 314:767–8. 10.1001/jama.2015.7762 26305643

